# Materiality and the mediating roles of eHealth: A qualitative study and comparison of three cases

**DOI:** 10.1177/20552076221116782

**Published:** 2022-08-01

**Authors:** Susanne Frennert, Lena Petersson, Mirella Muhic, Christofer Rydelfält, Veronica Milos Nymberg, Björn Ekman, Gudbjörg Erlingsdottir

**Affiliations:** 1 Department of Design Science, 5193 Lund University, Lund, Sweden; 2 Department of Health and Welfare, 3694 Halmstad University, Halmstad, Halland, Sweden; 3 Department of Informatics, 8075 Umeå University, Umea, Sweden; 4 Department of Clinical Studies, 5193 Lund University, Lund, Sweden

**Keywords:** eHealth, healthcare professionals, qualitative study, paradoxes

## Abstract

Against the backdrop of eHealth solutions increasingly becoming a part of healthcare professionals’ ways of doing care work, this paper questions how the solutions mediate the experience of healthcare professionals when deployed. We undertook a qualitative study of three eHealth solutions, conducting qualitative interviews with a diverse sample of 102 healthcare professionals from different care settings across the south of Sweden. Materiality and postphenomenology serve as analytic tools for achieving an understanding of the mediating roles of eHealth solutions. The analysis emphasises the mediating roles consisting of interrelated paradoxes: (1) changing and perpetuating boundaries between patients and professional groups, (2) (dis)enabling augmented information and knowledge processes and (3) reconfiguring professional control over work. This contribution provides critical insights into materiality as a category of analysis in studies on the deployment of eHealth solutions, as these technologies have both intended and unintended consequences for care work. Our study identified general positive consequences of all three solutions, such as the increased feeling of closeness to patients and colleagues over time and space; increased ‘understanding’ of patients through patient-generated data; and increased autonomy, due to the fact that asynchronous communication makes it possible to decide when and which patient to attend to. We also identified general unintended consequences of the solutions, such as maintenance of power relations maintained due to organisational structures and professional relations, disabled information and knowledge processes due to the lack of non-verbal clues, reduced professional autonomy due to technical scripts determining what data is collected and how it is categorised, and uneven workload due to the dependency on patient input and compliance.

## Introduction

In this article, we explore the mediating roles of three eHealth solutions in the care work experiences of healthcare professionals, using a postphenomenological perspective on the agency of technology in practice. eHealth technologies have since the beginning of the 21st century been considered a paradigm shift of healthcare towards improved healthcare. Eysenbach emphasised the importance of the ‘e’ in eHealth, which represents several es: efficiency, enhancing quality, evidence-based, empowerment, encouragement, education, enabling, extending, ethics and equity.^1^ In other words, the early concept of eHealth implied an advancement in healthcare provision by involving, engaging and empowering patients to partake in decisions about their health and care, which in turn would improve both the process and the quality of care. Today, when digital technologies have become the fabric of most parts of everyday life, there has been a significant uptake of eHealth solutions around the world.^2^ Research shows that the landscape of eHealth solutions is broad and comprises several different kinds of technologies with different purposes.^3^ According to Shaw et al.,^3^ three overlapping domains are prominent among eHealth solutions: (a) monitoring, tracking and informing about health; (b) communication and interaction between different stakeholders; and (c) collecting, managing and using health data. An eHealth solution can belong to one of these domains or contain elements from several domains.^3^

Research on eHealth has focussed on changed roles for healthcare professionals,^4–6^ the importance of alignment with care processes,^7–9^ impact of staff turnover,^10^ increased workload^11^ and/or decreased workload,^12–14^ changes in workflow^15–18^ and changes in professional values.^5,7,19^ Consequently, previous research shows that eHealth solutions influence the way healthcare professionals conduct their work and provide care. There are several challenges when implementing eHealth solutions whose purpose is to increase patient participation by changing the boundaries between patients and healthcare professionals.^20^ Professionals’ work is traditionally surrounded by boundaries, and constructing and maintaining the boundaries around their own area of knowledge is a fundamental part of professionals’ growth and development. Fournier^21^ highlights that changes in professional boundaries can lead to professions being reshaped and that boundaries change rather than disappear. In the daily work of an organisation, professional boundaries can be taken for granted, but in the event of changes in the form of the implementation of new technology, they can be made visible and questioned.^22^ Care and care work co-emerge through the production and reproductions of arrangements between patients, actions, values, care workers, institutions and eHealth solutions.^23–28^

Recent studies highlight a growing need to consider the materiality of eHealth solutions and technologies.^29–31^ Although eHealth solutions have been studied for years, there remains a gap in understanding of how the materiality of eHealth solutions affects care and care work.^18,32–34^ Leornadi^35^ defines materiality as ‘the arrangement of an artefact's physical and/or digital materials into particular forms that endure across differences in place and time and are important to users’. He argues that the material aspects of technology become materialised in a context and evolve in that context, that is, they are not fixed. Furthermore, these material aspects have different matters to different users.^35^ In this article materiality refers to the ways in which technologies affect care and care work, that is, the mediating roles of technology.

In this article, we explore the mediating roles of three different eHealth solutions from the perspectives of technology mediation and materiality. The results can be used to improve design practices of eHealth solutions in care. In particular, studies on the mediating roles of eHealth solutions through analysing relevant examples are the key to comparative analysis, that is, moving beyond the microfocus of individual case studies to a meta-analysis of several eHealth solutions. The results in turn can inform both practice and policy. The article is structured as follows. First, we discuss the theoretical underpinnings for our research, then we outline our cases consisting of three different eHealth solutions; thereafter, we present and discuss the analysis of our empirical data. In the last section, we consider the implications of our findings.

### Theoretical underpinning

Below we describe the theoretical underpinnings. In turn, we will explain the concepts of *technology mediation*^36–41^ and *materiality.*^42^

#### Materiality and technology mediation

According to the field of science and technology studies, technology is not value-free but embodies norms and values that dictate how it affords as well as hinders specific human actions and experiences.^41,43^ This implies that when added to the care process, technology may transform both the care process and its outcome.^25,26,28,44,45^ In practice there may thus be both gains and losses associated with the digital transformation of care, and the concept of affordance has been introduced to explain this phenomenon.^46^ Affordance refers to how the material aspects, or the design, of an artefact indicate to the user how the artefact can or should be used. For example, the material aspects of a hospital bed indicate that the user can lie on it, but a hospital bed can also be used to stand on; the hospital bed affords both. However, the context of use and norms direct the perceived affordance, that is, to lie in the hospital bed and not to stand on it.^46^ Here we apply a postphenomenological perspective. While phenomenology refers to the study of a phenomenon as we experience it,^47^ postphenomenology focuses on how technology mediates human experiences, actions and perceptions (i.e. technological mediation).^41^

The theory of technological mediation portrays the human–technology relation and builds on Don Ihde's philosophy of technology.^41^ According to Ihde,^39^ technology is intertwined in the way humans experience the world, and the material aspects of technology thus shape and mediate human experiences. This also implies that when the material aspects of digital technology are put into practice, it will shape the way work is organised and perceived. In other words, humans react and act on technology, which results in technology mediating how humans perceive, for example, what is considered as normal and abnormal, good health and sickness. The human–technology connection is thus relational, that is, inseparable, and constituted in and through human actions and material aspects of technologies.^48^ However, how technology shapes human experience is not fixed but depends on the human's interpretation and appropriation of the technology, as well as its material aspects and context of use.^37^ For example, a health monitoring system may be used for counting steps, keeping track of how many calories are burnt, measuring EKG and so forth to make the user aware of his or her health and lifestyle, but it may also be used for calorie intake restriction regimes (i.e. if one is suffering from anorexia).

#### Materiality and organisations

Leonardi and Barley^42^ highlight the complexity of studying the materiality of technology in organisations, as a specific technology used in an organisational context not only affords certain ways of working but also transforms the nature of work.^42^ Leonardi^35^ argues that we need to move beyond looking at how people perceive technology to focus on the interplay between non-human and human actors. Ihde,^39^ uses cultural hermeneutics as a concept for describing how human interpretations and appropriations of technology are built on cultural conditions. He argues that human–technology relations are not only individual but also collective. In other words, technologies are cultural artefacts, and the meaning technology is given is affected by cultural, historical and political values. Likewise, the material aspects of an eHealth solution play a crucial role in its mediation effect and performativity.^49^ In an organisational context technological mediation is constituted of both symbolic and material aspects. Meanings (discourse) and matters (materialised aspects) mutually affect the mediation role of an eHealth solution.

Thus, studying the mediating role of specific technologies provides us with knowledge about the effects of eHealth solutions in a certain context and how they change the very nature of care work. Next, we will examine the mediating role of three eHealth solutions, through three empirical cases.

### Three eHealth solutions

In this section, we introduce three studies on the effects of three different eHealth solutions on professionals’ work and work processes. First, *the Tablet,* a research study exploring an eHealth solution for monitoring patients who have home peritoneal dialysis (i.e. treatment for kidney failure). Second, *the Pod*, a research study investigating an eHealth solution for the care of patients who have been diagnosed with heart failure and patients with chronic obstructive pulmonary disease (COPD). Third, *the Flow*, a research study exploring an eHealth solution for digital pathways to primary care (see Table 1).

**Table 1.** Overview of the paths of traditional care versus digitally mediated care of the three eHealth solutions.

The tablet	The pod	The flow
Remote monitoring of patients with chronic kidney failure	Remote monitoring of patients who have heart failure or patients with COPD	Digital triaging of patients in primary care
*‘Traditional’ care*		
The patients need peritoneal dialysis, which is a treatment that filters wastes and water from the blood. The dialysis can take place either at home or at a clinic. At home, the patient or a staff member from the municipality records vital signs on a sheet of paper that is bought to the clinic during physical check-ups approximately once a month.	Calendar-driven care: the patient has regular physical appointments at the heart clinic, where healthcare professionals check for vital signs. The patient also receives lifestyle advice.	The patient phones the primary healthcare centre and gets a time when a nurse will call back. Over the phone, the nurse does a health assessment and preliminary diagnosis. The phone call results in either a physical appointment at the healthcare centre or the patient receiving medical advice for self-care.
	At the clinic, the healthcare professionals enter the collected data into the electronic health record.	
*Digitally mediated care*		
Patients are monitored remotely. The treatment at home is monitored by manually registering the weight of the dialysis bags and by measuring values from scales and blood pressure monitors. The values are automatically transmitted via Bluetooth technology to the tablet, and patients, healthcare professionals at the hospital and, in the case of assisted treatment, the staff at the municipality can get immediate access to the information. Either the patient handles her/his home dialysis four times/day or the patient has assisted treatment, in which a nurse from the municipality performs the home dialysis. The patient can, via an encrypted tablet, communicate with the care staff through video calls, chat messages and photos.	An individual profile is created for each patient, with personalised recommendations and critical values recorded in the digital application. The patient regularly reports various measurements such as blood pressure, weight, physical activity and an estimate of her/his mood. The patient is also given the opportunity to communicate remotely and asynchronously (via text messages) with the healthcare professionals at the heart clinic.	The patient initiates a digital contact with a healthcare centre through a digital platform. First, the patient fills in her/his medical history and symptoms. Based on the patient's symptoms, gender and age, questions are automatically generated.
The results are presented to healthcare professionals and patients via graphical presentations.	At the clinic, the patient's self-reported values are presented in the pod platform. An algorithm creates visualisations of the patients’ conditions and needs for prioritisation, and a priority overview of all patients’ medical status based on the self-reported patient values is displayed. Each time healthcare professionals look at a patient's values, a notification is automatically sent to the patient.	At the healthcare centre, a medical report based on the patient's input is generated, and each patient case is triaged to a specific healthcare professional (i.e. doctor, nurse, psychologist, etc.). Either the patient errand can be handled without further communication, or the patient is followed up with either a physical appointment at the healthcare centre, asynchronous communication via chat messages or synchronous communication via videoconferencing.
Patients can access the eHealth solution by a secure encrypted tablet. There is no need for any other digital verification (Bank ID).	Patients have to identify themselves with a digital verification (Bank ID) to be able to log into the eHealth solution.	Patients have to identify themselves with a digital verification (Bank ID) to be able to log into the eHealth solution.

The importance of this type of study is manyfold: *Firstly*, the healthcare sector has been criticised for the slow diffusion of digital technologies. The reasons given are often the lack of competence and/or interest among healthcare professionals,^50^ whilst the mediating role of healthcare technologies is seldom studied.^51,52^ *Secondly*, the healthcare sector has a high demand for change, motivated by difficulties in recruiting and keeping healthcare personnel as well as by the increasing cost of care.^53,54^ As the digital transformation is ongoing, it is important to understand the healthcare professionals’ perspective on the mediating role of technologies in care, as it may affect their job satisfaction.^55^ Thirdly, the three cases differ from each other (i.e. different development paths, aims and target users) but are surrounded by the same technological visions and imaginaries. Imaginaries here refer to a shared conception of the ideal healthcare system.^56^ The digital transformation of healthcare is portrayed as the solution to the increasing costs due to an aging population and increases in chronic conditions and represents a dominant sociotechnical imaginary.^56^ Apart from Sweden, many other countries have promoted and invested in this digital care imaginary (e.g. the United Kingdom, Australia, Denmark and more).^57–60^ In Sweden, the care imaginary has been manifested in three national eHealth strategies or visions, in 2006, 2010 and 2016^61–63^ which express similar values and arguments: innovation, patient-centred care, patient engagement, efficiency, availability, accessibility, equality and privacy.^64^ The latest eHealth vision even states that by 2025, ‘Sweden will be best in the world at using the opportunities offered by digitisation and eHealth, making it easier for people to achieve good and equal health and welfare, and to develop and strengthen their own resources for increased independence.’^63^ The three cases thus provide ideal settings to study the mediating role of eHealth solutions, as they both differ between themselves but still fit into the same Swedish organisational and cultural contexts during the same period of time. Next, we will describe the three different eHealth solutions.

### The Tablet

*The Tablet* was developed as a part of a collaborative research/development project between the university and the health tech department of the local region. The aim of the project was to develop digital support for healthcare professionals and patients in advanced care situations performed in patients’ homes (i.e. efficiency and enhancing quality). This particular part of the digital solution makes it possible for patients having dialysis in their homes to share their data digitally and simultaneously with the nurses and doctors at the dialysis department at the regional hospital. Traditionally, the patients would log their data manually on paper, which they then brought with them to their physical monthly visits to the hospital. Instead, the digital eHealth solution enables the medical staff at the hospital (mainly nurses) to continuously follow and monitor the patient's treatment. Also, the patient's data accumulates in the eHealth solution and can be visualised in diagrams that show development over time. Communication between the patient and the health care staff monitoring the process can be carried out through a digital chat or a video meeting. The data in the eHealth solution are not automatically transferred into the patient's electronic healthcare record (EHR), but some of the data are transferred manually by healthcare professionals.

### The Pod

*The Pod* was created by engineers who formed a company to develop the technology. The aim was to convey new technology into healthcare to enable new types of healthcare including remote monitoring and support of patients by digital communication, mediation of patient data and an algorithm that ranks patients according to their need for intervention*. The Pod* thus offers a possibility for healthcare workers to monitor patients in their homes and for patients to share their health data and be monitored. The eHealth solution enables patients to enter their data and chat with healthcare professionals. In practice, the patients use their own computers, smartphones or tablets to reach the homepage of the service. The patients need the digital verification Bank ID to identify themselves when entering the service. In the eHealth solution, they enter their vital parameters, which may vary between patients. These parameters are compared to set intervals for every vital parameter so that the algorithm can evaluate and signal when the parameters fall outside the set interval. The patients who are invited to the eHealth solution have some type of chronic illness, for instance, heart failure or COPD. *The Pod* is implemented primarily by hospital departments, but also by healthcare centres supporting COPD patients. Their aim is to give patients with chronic illnesses more personalised/relational care and contact with the caregiver (i.e. patient empowerment). The purpose is to improve the patients’ health and thereby keep them from rehospitalisation (i.e. cost efficiency). The data in the eHealth solution are not automatically transferred into the patient's EHR, but some of the data are transferred manually by healthcare professionals.

### The Flow

*The Flow* was developed by general practitioners who perceived, through their own work, a need for digital patient management in Swedish primary care. They started a company and developed a platform to improve medical quality, resource utilisation and patient experience (i.e. efficiency and patient empowerment). *The Flow* has been implemented by several private and public primary healthcare centres in Sweden. It is also used in other European countries such as Norway, the Czech Republic, Poland and the United Kingdom. In contrast to traditional Swedish primary care routines, *the Flow* enables digital patient contact (Table 1). Communication and patient meetings take place either synchronously or asynchronously in the form of digital (video or chat) or physical meetings with different categories of healthcare professionals (Table 2). The data in the eHealth solution are not automatically transferred into the patient's EHR, but some of the data are transferred manually by healthcare professionals.

**Table 2.** Description of the eHealth solutions.

Functionalities and architecture	The tablet	The pod	The flow
Automated tracking of patient data	Yes – weight and blood pressure from provided equipment via Bluetooth	No – requires patient input	No – requires patient input
Possibility for chat messages	Yes	Yes	Yes
Possibility for video consultations	Yes	No	Yes
Possibility for sharing pictures	Yes	No	Yes
Data visualisations of patient data	Yes	Yes	Yes
Requires digital verification with Bank ID	No	Yes	Yes
Requires private equipment, i.e. smart phone, tablet or computer	No – the caregiver provides the necessary scale and tablet	Yes	Yes
Integrated with electronic healthcare record (EHR)	No	No	No

### Methods

The data derived from a qualitative study of healthcare professionals (i.e. nurses, general practitioners, medical administrators, psychologists, and line managers) experiences of using three eHealth solutions. The study was designed and conducted by a multidisciplinary team of researchers. Data were collected over a 36-month period (2020–2022). Four researchers (SF, LP, GE, ML) conducted 102 semi-structured interviews^65^ with a variety of healthcare professionals at four primary healthcare centres, seven hospital departments and one heart failure clinic. Participants were recruited via a combination of purposive^66^ and snowball sampling.^67^ Potential participants received an email describing the study and why they were being invited to participate. We recruited nurses, physicians, medical administrators, psychologists and line managers to capture both depth and breadth^68^ of care work experiences when using the eHealth solutions. The recruitment of interviewees was a dynamic process^68^ that continued until few new viewpoints emerged. The interviews took place at the healthcare professional's workplace or via phone or videoconferencing. The semi-structured interviews^65^ were conducted as a conversation with a specific aim (i.e. understanding the lived experience of care work while using the eHealth solution). The interviewer followed a semi-structured guide,^69^ and interviewees were asked to describe situations in which they used the eHealth solution (i.e. the *Pod*, the *Tablet* or the *Flow*) and to talk about their experiences of care work when using the eHealth solution in different situations and in relation to their professional role, patients and colleagues, for example: Have you had situations in which the eHealth solution has been really helpful? Have you had situations where problems arose? Can you give any examples of when the eHealth solution has affected your workload? Can you give any examples of when the eHealth solution has affected patient relations? The interviewer used probing questions such as, How does it make you feel? Could you please expand? What do you mean by XX? The interviews lasted 30–75 min and were audio recorded and transcribed verbatim by a transcription agency. Sampling and data collection are summarised in Table 3.

**Table 3.** Overview research settings.

	The tablet	The pod	The flow
*Settings*	Five hospital departments of nephrology and municipalities where patients with homecare use the eHealth solution	One heart failure clinic, two hospital departments of cardiology and a primary care centre	Three primary care centres
*Participants*	Nurses, physicians, care administrators, municipal assistant nurses and municipal nurses	Nurses, physicians, line managers	Nurses, general practitioners, line managers, psychologists, care administrators
*Method*	30 semi-structured interviews	19 semi-structured interviews	53 semi-structured interviews
*Duration*	6 months	2 years	2 years and ongoing

### Data synthesis

The research is based on a qualitative and interpretive approach with regard to both data collection and method of analysis,^70,71^ focussing on the participants’ words and narratives on the mediating role of the three different eHealth solutions. As we used an interpretive approach,^71^ we were not interested in predicting behaviour but instead aimed to examine the lived experiences of doing care work using the eHealth solutions, that is, the mediation effect on care and care work.^72^ Six of the seven authors have extensive training and practice in qualitative methods. The first author, who has a background in human–computer interaction, the second author, who has a background in pedagogy, and the last author, who has a background in organisational studies, coded the data. The data analysis was organic and followed a cyclical process, inspired by Braun and Clarke.^73,74^ The first step was data familiarisation. The first author, who conducted most of the interviews on the *Flow,* read and re-read all the transcripts to get a sense of the whole. The second author, who had conducted the interviews on the *Tablet* and the *Pod*, read and re-read all the transcripts for the *Tablet* and the *Pod*. The last author, who had conducted the interviews for the *Pod,* read and re-read transcripts from all the cases. The three authors discussed their initial understandings and interpretations of the data, focussing on how the mediating roles of the three eHealth solutions were elaborated on by the healthcare professionals. During this stage, it became apparent that there were general issues with the ways in which the three different eHealth solutions mediated work. It was decided to do a cross-case analysis^75–77^ to dig deeper into whether or not general insights about the mediating effects of these solutions could be derived. The cross-case analysis is a method for an in-depth examination of similarities and differences across cases, aiming to explore empirical generalisability.^76^ The data were coded by hand. First, we used open coding, that is, we read all the transcripts and wrote headings, comments and reflections in the margins.^70^ Thereafter, the codes were organised in a table, one table for each case. The codes were then compared for similarities and differences, first within each case and then across the three cases, after which they were grouped.^71^ Next, the potential themes were reviewed, defined and named by the first, second and last author. In each theme, we found paradoxes surrounding the mediating roles of the eHealth solutions. The co-authors, with backgrounds in organisation, informatics, computer science, psychology and medicine, checked and discussed the analysis. Although the team members have different backgrounds and perspectives, all reached an agreement on the essence of the themes. To examine the credibility of the interpretations, the themes were presented, on several occasions, to academic colleagues and healthcare professionals.^78^ During this iterative process, we modified and clarified the themes and cultured our theoretical underpinning. The result of this iterative analysis process is presented in this paper.

### Findings

In this section, we present our findings. Through exploring the lived experiences, among healthcare professionals, of working with three eHealth solutions, we found paradoxes attached to the mediating roles of all three solutions. The paradoxes concerned three different themes: (1) changing and perpetuating the boundaries between patients and professional groups, (2) (dis)enabling augmented information and knowledge processes and (3) reconfiguring professional control over work. Below, we outline the themes. The quotations used in text are those that best illustrate the themes. The names are pseudonyms.

#### Changing and perpetuating the boundaries between patients and professional groups

When participants were asked to compare their practices with the eHealth solutions to those before the deployment of the technologies, an almost uniform response revealed that the eHealth solutions had social consequences for their work. Most of our participants highlighted that the interaction with patients through the eHealth solutions changed boundaries with patients; the eHealth solutions mediated closeness with patients due to the increased amount of patient-generated data and the ease with which they could communicate with patients through the chat function compared to trying to reach patients through phone and postal mail. The healthcare professionals could react to patient input through text messaging (chat) with the patients and asking follow-up questions. As a result, most of the participants felt closer to the patients:

The best thing is that I get closer to the patients … the ability to send and receive pictures … If you want to run a video meeting, you press the video meeting button. (Peter, a physician using *the Flow)*

The quote illustrates the ease the physician felt in the ability to contact patients through *the Flow*. The argument was recurrent during several interviews. For example, as one of the nurses, Maria, using *the Tablet* related:

It is also faster communication when you can chat with each other or send a message. Before that you could call the patient and if they did not answer, then you had to try to call again and again. Now I can just send a message, and the patient will see it at some point during the day, so to speak.

The participants also talked about the ease with which they could communicate with colleagues via the interface of the eHealth solutions. For example, in *the Flow*, they had a message board that was only visible to healthcare professionals and not to patients. As they could see the different patient errands, they could also keep up to date with the work others had done in certain patient errands:

Yes, that you get a better insight into each other's everyday lives …. You can start a case and then something new is added, and then you have to transfer it to the doctor and then you get it back …. It is good for both of us, both groups [nurses and physician], that you learn from each other. (Anna, a nurse using *the Flow)*

The extract above illustrates what many participants mentioned; they felt closer to other professional groups, as the eHealth solutions gave insights into others’ work and, as result, they learned from each other. Spatial boundaries between the clinic and the patients’ homes as well as between different clinics seem to dissolve with the use of the eHealth solutions. As the eHealth solutions enabled asynchronous communication, time boundaries also dissolved; healthcare professionals could reach out to patients and professional groups when they found time in their schedule. Likewise, patients could contact healthcare professionals when they were able to or wished to.

On the other hand, the eHealth solutions also perpetuated the boundaries with patients. Although the patients could use the eHealth solutions to contact healthcare professionals digitally around the clock, the healthcare professionals would only attend to patient-generated data and patient errands if time allowed and during the daytime when they worked. One aim of deploying the eHealth solutions was to become more accessible to patients. However, the working hours for the healthcare professionals were not changed alongside the deployment of the eHealth solutions. As a result, the patients received feedback during the daytime, regardless of when they contacted the healthcare professionals. If the healthcare professionals had meetings, physical visits or other activities, the patient-generated data were not attended to. Some participants felt that patients had an understanding of always being looked after due to the use of the eHealth solutions (i.e. *the Tablet* and *the Pod*), but in practice that was not the case because of pre-existing organisational structures.

The eHealth solutions also perpetuated the boundaries between professional groups, in particular between nurses and physicians. Before deploying the eHealth solutions, the nurses were responsible for organising and collecting patient data (e.g. history taking, diagnostic reasoning, physical examinations, follow-up phone calls, etc.), while the doctors were responsible for deciding the right diagnosis and the treatment plan for patients. When introducing eHealth solutions to the care settings, the nurses became responsible for the day-to-day handling of patient errands and patient-generated data stipulated by the eHealth solutions, while the doctors only seldom used the eHealth solutions to keep track of patients. As such, the professional boundaries between the nurses and physicians were perpetuated. As one of the nurses, Karin, using the Pod, explained:

So, the doctors are not very involved in the Pod … They do not work so much with it … they are not in and check and so on. They may at some point ask how a patient is doing, how they are feeling and so on.

As illustrated by the quotation above, the lived experience of nurses was that they were responsible for the day-to-day engagement with patients through the eHealth solution, while the physicians’ work was not affected to the same degree by the deployment of the eHealth solutions. As this theme of the ‘boundaries paradox’ has shown, in practice, the use of the three eHealth solutions both created closeness to patients and professional groups and perpetuated power relations.

#### (Dis)Enabling augmented information and knowledge processes

Here we use a parenthetical prefix, (dis), to highlight that the eHealth solutions both enabled augmented information and knowledge processes and disabled them.

Many of the participants praised how the eHealth solutions visualised data through graphs and visual representations. The visual presentation of data enabled increased possibilities, compared to before the use of the eHealth solutions (Table 2), to understand and grasp patterns and trends in the health status of patients. The healthcare professionals working with *the Tablet* automatically received the patients’ daily weight and EKG results; the healthcare professionals working with *the Pod* received information about their patients’ blood pressure, weight and physical activity, and an estimate of her/his mood; the healthcare professionals working with *the Flow* received automated triaging based on patients’ own words about their health and illness (Table 1). The solutions (i.e. *the Tablet* and *the Pod*) made it possible to follow how different medical interventions impacted the patients and to follow the progress over time. The increased amount of patient-generated data, as well as its visual representations, became foundations for decision-making, helping to prioritise which patients needed direct attention and which patients could wait. For example, when logged in, the healthcare professionals who worked with *the Flow* were met with a graphical overview containing each patient case being triaged and a heading explaining why the patient was seeking care assistance. One of the line managers, Ulla, using *the Flow*, explained it as following:

When the patient calls us on the phone, it is a blank slate. We need to start from the beginning and ask questions and follow-up questions. Before we can prioritise that patient, it will take some time, while in *the Flow*, it becomes very easy for us to prioritise, because history-taking has already been done before it comes to our attention. So, therefore, it is very easy for us, that the patient uses *the Flow* instead of the telephone.

The line manager talked about how the use of *the Flow* had simplified the prioritising of patients compared to answering patient errands over the phone. When answering the phone, the nurse initially had very few details and needed to ask questions in order to comprehend the patient and the patient's errands. When using *the Flow*, the patient responded to automated questions, and the nurse received a resulting medical report. The nurse could then read the report and also look up the patient's healthcare record before attending to the patient. As a result, the nurse already had a depiction of the patient and the patient's needs before the communication started. Similarly, the healthcare professionals using *the Tablet* and *the Pod* received patient-generated data that were graphically displayed by the eHealth solutions. A physician, Ola, using *the Tablet*, stated the following:

Yes, it significantly facilitates the monitoring of the patient, of course. It makes it very, very much easier for us to see in almost real time what is happening to the patient. We can find out what the patient weighs; we can find out blood pressure, heart rate.

The physician highlighted that the automated tracking of patient data and the data visualisation by the eHealth solution made it easier to monitor patients, and as a result, to decide the next step in the treatment. Furthermore, the healthcare professionals found comfort in receiving an increased amount of patient-generated data, as they could communicate the data through the eHealth solutions with other healthcare professionals not only at their own workplace but also across workplaces. Care work is characterised by teamwork; the healthcare professionals worked with various categories of healthcare professionals within the clinic and also across clinics and with municipal healthcare professionals. The communication between them took place orally, in meetings or by phone, and in writing, in emails, by fax or through the healthcare records. Although the eHealth solutions enabled data sharing similar to the healthcare records, the eHealth solutions were favoured, as they had data visualisations and graphical interfaces, and consisted of the patient's input along with the healthcare professionals’ notes. The eHealth solutions were also seen as augmenting information and knowledge processes, as the data visualisations enabled substitute healthcare professionals to take care of patients that they normally did not care for. As one of the physicians, Martin, who used *the Tablet*, said:

It is also easy to form an idea of where the fault lies. Otherwise, if I do not know the patient, then I have to book a meeting with the patient …. She must bring all the papers and I have to go through them. And if it becomes something urgent, then I can just go into the Tablet and check what changes have taken place in the blood pressure or fluid balance …. So it is easier too, to fix urgent problems for patients that I have not known from the beginning.

Not only were the eHealth solutions perceived as augmenting information and knowledge processes for the healthcare professionals, but they (i.e. *the Tablet* and *the Pod*) were also perceived by the healthcare professionals as facilitating for patients an increased understanding of their illness and also serving as a pedagogical tool to help patients understand the impact of their actions on their health, for example, how alignment with medical treatment affects their health and what impact exercise and eating habits have.

Thus, most healthcare professionals argued that the eHealth solutions mediated augmented information and knowledge processes and acknowledged that the use of the eHealth solutions also led to fewer physical meetings and communication by phone. However, this in turn also resulted in (dis)enabling information and knowledge processes, as they needed to rely on, to a higher degree than before the deployment of eHealth solutions, on quantified data and patients’ input. As one of the physicians, Berit, using the *Flow*, explained:

You only see text and measurements. Body language is important for an adequate medical analysis. During physical visits I can see if the patient looks comfortable or uncomfortable, if she is in pain or pale, if she is attentive or has trouble focussing and more. There are more dimensions that can be taken into the medical analysis when you physically meet compared to digital communication.

Similar to Berit's reflection, many of the participants mentioned that through phone calls with patients and in physical meetings, they could take into account non-verbal clues (i.e. body language, breathing, swelling, etc.), but through the eHealth solutions non-verbal clues were diminished.

#### Reconfiguring professional control over work

The healthcare professionals thought that the eHealth solutions had both a positive and a negative effect on their professional control and autonomy. On one hand, the eHealth solutions increased autonomy, as the healthcare professionals were in charge of deciding which patient errand to attend to and when:

The advantage is that you get measures and values directly from the patient, and I can take care of them during the day when I have the time to take care of them. I’m not dependent on sitting at the phone between eight and nine, for example, but I might be able to do it at half past nine instead, go in and look at my patients’ measures and values. (Kerstin, a nurse using *the Tablet*)

Most of the healthcare professionals, as in this extract, highlighted that the asynchronous communication through the eHealth solutions increased their autonomy. Patient-generated data also made it easier to plan and be in control when they had physical patient visits, as they were already aware, through the eHealth solution, of the patient's state of health. On the other hand, the design of the eHealth solutions governs which data are collected and how they are categorised:

In *the Flow* the patients completely control what they want to focus on … or *the Flow* has this automated questionnaire which I assume they [the patients] get for different symptoms … and then the triaging is controlled by the questions *the Flow* asks, so to speak, whereas in telephone contact, then it is I who control the questions asked and identify the problem. (Birgitta, a nurse using *the Flow*)

The nurse emphasised that before the introduction of *the Flow* she oversaw triaging the patients, but now the eHealth solution automatically triaged the patients based on their inputs. Sometimes, this was a cause of concern, as the healthcare professionals had an impression that some patients exaggerated symptoms and overused the possibilities of digital contacts:

The phone was somehow a filter; we could not be reached at any time, anyway. And now it's very easy. When a patient wants to, she can contact us around the clock …. There are patients who contact us all the time, almost every day, or several times a day, and then we have the responsibility to get back to the patient, even if the matter is vague or irrelevant, and it becomes unsustainable. (Stina, a nurse using *the Flow*)

The nurse explained that there were patients who contacted them daily, which in turn increased their burden of work, as they received more patient errands. Before deploying *the Flow*, the long phone queues and phone hours worked as a gatekeeper. Moreover, access to care by telephone was characterised by arbitrariness in that the severity of the patient's condition did not determine who got through. Now, *Flow* worked as a gatekeeper to people who were not digitally literate. The healthcare professionals working with the eHealth solutions for monitoring (*the Tablet* and *the Pod*) did not experience patients overusing the eHealth solutions, but they acknowledged that for the healthcare professionals to feel in control of the care process, their patients, in turn, needed to be active and input data; without patient-generated data, they were left in limbo regarding the patient's state of health. As a result, healthcare professionals became dependent on patient input. Some days, in all the three cases, they received little or no urgent patient data to attend to, while other days they received a vast amount:

You can go in one day, and then all the patients really feel just fine and there are no urgent patient data. Nobody has anything. Then you can go in another day, and it's like eight patients who are completely marked in red. Everyone feels bad. And then you have … more things to do, but another day, it's like zero to do. It's a bit difficult to plan your workday in advance. (Julia, a nurse using *the Pod*)

The nurse reported that the workload changed from day to day, depending on patient input; this made it difficult to be in control and to plan ahead. Thus, the eHealth solutions decreased healthcare professionals’ control over their work, due to dependency on patients’ input and material aspects of the eHealth solution and on the embedded scripts by which data were collected and how they were categorised. At the same time, they increased healthcare professionals’ control of their work, due to the solutions’ enabling asynchronous communication in which the healthcare professionals could decide when, and which patient, to prioritise, and due to an increased amount of information about the patient, which gave them more control in physical meetings and eased decision-making.

### What can we learn from these three cases?

The focus of postphenomenology is to understand the role technology plays in human actions and experiences.^39,41^ In this article, we have studied three different eHealth solutions and their mediating role in care and care work. Our cross-case analysis shows that eHealth solutions are not mere tools that can identify and predict patients’ health and illness by standardised and deductive methods.

*First*, our analysis brings about contradictory translations of the boundaries between patients and professional groups. All three eHealth solutions were developed and deployed to better organise care work around patients. For *the Tablet* and *the Pod*, this entailed monitoring patients through patient-generated data at a distance, while for *the Flow*, this entailed digital access to the healthcare centre for common illnesses and conditions among citizens. Recent work, in particular by Piras and Miele,^79^ has focussed on digital intimacy. They argue that remote monitoring and exchange of text messages between patients and healthcare professionals can foster a greater intimacy between patients and healthcare professionals than traditional care. The level of intimacy depends on the quantitative and qualitative communication between patients and healthcare professionals.^79^ While we would agree with Piras and Miele, that the eHealth solutions studied increased intimacy with patients, we also observed that the eHealth solutions studied increased intimacy among professional groups, as they could follow each other's work and communicate through the solutions. However, in our cases, increased intimacy with patients and professional groups through the use of eHealth solutions paradoxically coexisted with prevailing power relations. The healthcare professionals were dominant in deciding when and which patient to respond to, while the patients could decide which data to share. Nimmon and Stenfors-Hayes^80^ highlight that the ideal in person-centred care of shared power between patients and healthcare professionals has yet to be realised. The empowerment of patients through the eHealth solutions studied was limited due to organisational structures (i.e. working hours and task priorities) and professional power (i.e. patient requests were subordinated to the healthcare professionals’ time to attend to patient errands). As such, the eHealth solutions acted as normative devices^81^ delegating power to healthcare professionals over patients. Similarly, our analysis shows that professional power relations were manifested by the use of the solutions; the nurses were responsible for the day-to-day handling of the solutions and the patient-generated data, while physicians used the solutions and data for decision-making. The increased responsibility of the eHealth solutions for nurses may in turn lead to increased invisible work (i.e. the day-to-day handling of the solutions), which often affects women more than men,^82,83^ as well as work associated with lower status (nurses) than higher status (physicians).^84^

*Second*, a focus on materiality rather than the positivistic viewpoint that an eHealth solution, correctly designed, programmed and used, will lead to a more efficient care process draws attention to the way cultural values and material aspects of technology shape and reshape the meaning and experience of patients’ health and illness.^85^ In our three cases, all three eHealth solutions used graphical data visualisations to describe the patient-generated data to healthcare professionals. The healthcare professionals greatly appreciated this feature, as it mediated augmented information and knowledge processes. The professionals claim that data visualisations amplified a patient's state of health over time as well as highlighting, through colours, who needed urgent attention. As such, the patient-generated numerical data provided opportunities for healthcare professionals to make sense of their patients’ conditions without physically meeting with them, that is, they extended their gaze and range of actions.^45^ This in turn, was perceived as enabling the healthcare professionals to handle patients of their colleagues, as they ‘only’ needed to interpret data and patterns in the eHealth solution in order to decide the next step. However, as the data are decontextualised, healthcare professionals need to recontextualise the data to interpret them.^86^ This requires tacit knowledge and judgement.^87,88^ Torenholt and Tjörnhöj^86^ highlight that the recontextualisation of data requires, in addition to skills and training, available time and resources. Our findings do not elucidate whether the participants had the right skills, training, available time and resources required, but many of them acknowledged that the non-verbal clues (i.e. body language, breathing, swelling, etc.) they often relied on and found important were diminished through the use of the eHealth solution.

*Third*, a conceptualisation of professional control in terms of postphenomenology enables us to analyse how human–technology relations matter in shaping healthcare professionals’ control over their work. Jhala and Menon^89^ argue that asynchronous communication in healthcare increases workflow efficiency, which in turn will increase patient safety and quality of care. All three eHealth solutions studied enabled asynchronous communication. Our findings do not illuminate increased workflow efficiency but reveal that asynchronous communication mediated professional control and autonomy, as the healthcare professionals could get an understanding of patient errands before contacting patients and could decide when to contact patients. In contrast to Jhala and Menon's study,^89^ our findings indicate that the workflow may be interrupted, as the workflow is dependent on active patients and their data input. The eHealth solutions redistributed responsibility for data entry to patients; it became the patients’ duty, and it was expected that the patients had the skills and knowledge required for patient data input. As a result, increased professional control over work paradoxically coincides with decreased professional control over work and increased dependency on patients. Furthermore, our findings also indicate that the professional control over work is decreased due to the eHealth solution design concerning which data to collect, and how these are categorised and visualised. Hoeyer and Wadmann^90^ argue that data can become prime means for care and care work. This may result in decreasing professional control when shifting the focus on clinical practice and patient care towards data exchange and datafication,^90^ thus shifting the power dynamics from the healthcare professionals towards eHealth developers and the ones in charge of the implementation.

This study contributes to unwrapping the black boxes of eHealth solutions, illustrating how the materiality of three healthcare solutions is shaping care work. The analysis shows that eHealth solutions are not merely neutral tools that automatically lead to efficiency and enhanced quality but are a transformative part of care and care work; that is, eHealth solutions and care work are co-constituted.^41,43,85^ This implies that achieving the several e's in eHealth in practice, namely, efficiency, enhancing quality, evidence-based, empowerment, encouragement, education, enabling, extending, ethics and equity,^1^ is more multifaceted than just deploying eHealth solutions. It requires sensitivity to materiality,^35^ as the deployment of new technology and technological change is not always linear or progressive, often transforming human actions and experiences in unanticipated and unintended ways.^45,91,92^ Our analysis shows how the eHealth solutions both supported and enhanced care work but also unsettled and compromised it. The dual effects of eHealth solutions are shaped by material aspects and context of use, and conditioned by organisational, political and cultural dimensions.^93^

#### Strengths and limitations

The strength of the article is that it is based on three studies of the effects of eHealth technology on care professionals’ work. The combination of the three cases makes it possible to make comparisons and cross-case analyses, which is unusual in this field of research, and to draw more far-reaching conclusions than a single case study would allow. The theoretical perspective of postphenomenology and materiality also enables critical analysis of the possibilities and limitations that technologies offer, which provides important insights into the multifaceted and often unintentional effects of technology use in practice.

The professionals interviewed had different competencies and experience, with varied perspectives of eHealth solutions in primary care. This increases the generalizability of our results and is a strength of the study. However, our findings can only be applied in a Swedish context and related to the described eHealth solutions, and this is a limitation. None of the researchers who conducted the interviews are healthcare workers but had a preunderstanding of the topic from previous research. This increases the reflexivity of our findings, which is also a strength.

The professionals’ perspective highlighted in the article is also important for an understanding of how eHealth solutions affect the work of professionals. At the same time, the empirical material has its limitations in that it provides a rather one-sided view, albeit important, on the part of the professionals. Further research is needed on different eHealth solutions, as they provide different insights and opportunities for comparison. The patient perspective is, of course, important, and we look forward to studies where the experiences of professionals and patients can be compared together, with analyses based on other theoretical perspectives that can complement and contrast those used in this article.

## Conclusion

This study explores healthcare professionals’ lived experience of using three different eHealth solutions. Our cross-case analysis shows interrelated paradoxes in their mediating roles: on one hand, the eHealth solutions increase closeness between the patients and professional groups across space and time, augment information and knowledge processes through data visualisations of patient-generated data and increase professional control and autonomy as the asynchronous communication and patient-generated data permit the healthcare professionals to decide when, and which patient, to attend to. On the other hand, the solutions perpetuate boundaries between patients and professional groups due to organisational structures and sustained power relations, disable information and knowledge processes due to the lack of non-verbal clues and decrease professional control due to the dependency on patient input and the embedded scripts of the technological solutions concerning which data to collect and how they are categorised. These findings suggest the need for careful design and implementation strategies for eHealth solutions in order to achieve the ambitions of empowerment, efficiency and enhancing quality.^1^

## References

[1] Eysenbach G . What is eHealth? J Med Internet Res 2001; 3: e20. 11720962 10.2196/jmir.3.2.e20 PMC1761894

[2] van der Kleij RM Kasteleyn MJ Meijer E , et al. SERIES: eHealth in primary care. Part 1: concepts, conditions and challenges. Eur J Gen Pract 2019; 25: 179–189. 31597502 10.1080/13814788.2019.1658190 PMC6853224

[3] Shaw T McGregor D Brunner M , et al. What is eHealth (6)? Development of a conceptual model for eHealth: qualitative study with key informants. J Med Internet Res 2017; 19: e8106. 10.2196/jmir.8106 PMC5676031 29066429

[4] Hunting G Shahid N Sahakyan Y , et al. A multi-level qualitative analysis of telehomecare in Ontario: challenges and opportunities. BMC Health Serv Res 2015; 15: 1–15. 26645639 10.1186/s12913-015-1196-2 PMC4673764

[5] Sølling IK Carøe P Mathiesen KS . Development and implementation of IT require focus on user participation, acceptance and workflow. In: *Nursing Informatics* 2014, pp.219–226. 24943547

[6] Grünloh C Myreteg G Cajander Å , et al. Why do they need to check me?” patient participation through eHealth and the doctor-patient relationship: qualitative study. J Med Internet Res 2018; 20: e8444. 10.2196/jmir.8444 PMC5789160 29335237

[7] Nielsen JA Mathiassen L . Interpretive flexibility in mobile health: lessons from a government-sponsored home care program. J Med Internet Res 2013; 15: e236. 24172852 10.2196/jmir.2816 PMC3841343

[8] Nazi KM . The personal health record paradox: health care professionals’ perspectives and the information ecology of personal health record systems in organizational and clinical settings. J Med Internet Res 2013; 15: e2443. 10.2196/jmir.2443 PMC3636319 23557596

[9] Andersen TO Bansler JP Kensing F , et al. Aligning concerns in telecare: three concepts to guide the design of patient-centred eHealth. Comput Supported Cooperative Work (CSCW) 2019; 28: 1039–1072.

[10] Barrett M Larson A Carville K , et al. Challenges faced in implementation of a telehealth enabled chronic wound care system. Rural Remote Health 2009; 9: 1–9. 19705955

[11] Öberg U Orre CJ Isaksson U , et al. Swedish Primary healthcare nurses’ perceptions of using digital eHealth services in support of patient self–management. Scand J Caring Sci 2018; 32: 961–970. 28960451 10.1111/scs.12534

[12] Gray CS Gill A Khan AI , et al. The electronic patient reported outcome tool: testing usability and feasibility of a mobile app and portal to support care for patients with complex chronic disease and disability in primary care settings. JMIR Mhealth Uhealth 2016; 4: e5331. 10.2196/mhealth.5331 PMC4911509 27256035

[13] Davis MM Currey JM Howk S , et al. A qualitative study of rural primary care clinician views on remote monitoring technologies. J Rural Health 2014; 30: 69–78. 24383486 10.1111/jrh.12027 PMC3882331

[14] Versluis A Schnoor K Chavannes NH , et al. Direct access for patients to diagnostic testing and results using eHealth: systematic review on eHealth and diagnostics. J Med Internet Res 2022; 24: e29303. 35019848 10.2196/29303 PMC8792777

[15] Embi PJ Weir C Efthimiadis EN , et al. Computerized provider documentation: findings and implications of a multisite study of clinicians and administrators. J Am Med Inform Assoc 2013; 20: 718–726. 23355462 10.1136/amiajnl-2012-000946 PMC3721152

[16] Janols R Lind T Göransson B , et al. Evaluation of user adoption during three module deployments of region-wide electronic patient record systems. Int J Med Inf 2014; 83: 438–449. 10.1016/j.ijmedinf.2014.02.003 24630924

[17] Weber JH Kuziemsky C . Pragmatic interoperability for ehealth systems: the fallback workflow patterns. In: *2019 IEEE/ACM 1st International Workshop on Software Engineering for Healthcare (SEH)* 2019, pp.29–36. IEEE.

[18] Frennert S Erlingsdóttir G Muhic M , et al. Embedding and integrating a digital patient management platform into everyday primary care routines: qualitative case study. JMIR Formative Res 2022; 6: e30527. 10.2196/30527 PMC8905477 35191845

[19] Wolferen P . The needs and wishes of healthcare professionals regarding the use of an eHealth technology. Twente, Netherlands: University of Twente, 2019.

[20] Petersson L . Paving the way for transparency: how eHealth technology can change boundaries in healthcare. Lund, Sweden: Lund University, 2020.

[21] Fournier V . Boundary work and the (un) making of the professions. Profesisonalism, boundaries and workplace. In: Malin N (ed) Professionalism, boundaries and the workplace. London: Routledge, 2000, pp.67–86.

[22] Lindberg K Styhre A Walter L . Assembling health care organizations: practice, materiality and institutions. Basingstoke: Springer, 2012.

[23] Pols J . Wonderful webcams: about active gazes and invisible technologies. Sci Technol Hum Values 2011; 36: 451–473.

[24] Pols J . Good relations with technology: empirical ethics and aesthetics in care. Nurs Philos 2017; 18: e12154. 10.1111/nup.12154 27758077

[25] Oudshoorn N . Physical and digital proximity: emerging ways of health care in face-to-face and telemonitoring of heart-failure patients. Sociol Health Illn 2009; 31: 390–405. 19144084 10.1111/j.1467-9566.2008.01141.x

[26] Mol A . The body multiple: ontology in medical practice. Durham: Duke University Press, 2002.

[27] Gómez DL Criado TS . Civilising technologies for an ageing society? The performativity of participatory methods in socio-gerontechnology. In: Peine A Marshall BL Martin W Neven L (eds) Socio-gerontechnology: interdisciplinary critical studies of ageing and technology. London: Routledge, 2021, pp.85–99.

[28] Greenhalgh T Wherton J Papoutsi C , et al. Beyond adoption: a new framework for theorizing and evaluating nonadoption, abandonment, and challenges to the scale-up, spread, and sustainability of health and care technologies. J Med Internet Res 2017; 19: e367. 29092808 10.2196/jmir.8775 PMC5688245

[29] Leonardi PM . Digital materiality? How artifacts without matter, matter. First Monday 2010; 15: 5–10.

[30] Svensson I Gluch P . Materiality in action: the role of objects in institutional work. Construct ManageEcon 2022; 40: 41–55.

[31] Carboni C Wehrens R van der Veen R , et al. Conceptualizing the digitalization of healthcare work: a metaphor-based critical interpretive synthesis. Soc Sci Med 2022; 292: 114572. 34839086 10.1016/j.socscimed.2021.114572

[32] Almeida T . New materiality in intimate care. Textile Intersections 2019; 5: 1–7.

[33] Esmonde K . Training, tracking, and traversing: digital materiality and the production of bodies and/in space in runners’ fitness tracking practices. Leisure Studies 2019; 38: 804–817.

[34] Keen J Ruddle R Palczewski J , et al. Machine learning, materiality and governance: a health and social care case study. Inf Polity 2021; 26: 57–69.

[35] Leonardi PM . Materiality, sociomateriality, and socio-technical systems: what do these terms mean? How are they different? Do we need them. In: Leornadi PM Nardi BA Kallinikos J (eds) Materiality and organizing: social interaction in a technological world. Oxford: Oxford University Press, 2012, pp.25–48.

[36] Verbeek P . Beyond interaction: a short introduction to mediation theory. ACM Interactions 2015; 22: 26–31.

[37] Verbeek P-P . Moralizing technology: understanding and designing the morality of things. Chicago: University of Chicago Press, 2011.

[38] Verbeek P-P . Don Ihde: the technological lifeworld. In: American Philosophy of technology: The empirical turn. Indiana: Indiana University Press, 2001, pp.119–146.

[39] Ihde D . Technology and the lifeworld: From garden to earth. Bloomington: Indiana University Press, 1990.

[40] Ihde D . Technology and prognostic predicaments. AI Soc 1999; 13: 44–51.

[41] Verbeek P-P . Toward a theory of technological mediation. *Technoscience and postphenomenology: The Manhattan papers* 2015: 189.

[42] Leonardi PM Barley SR . Materiality and change: Challenges to building better theory about technology and organizing. Inf Organ 2008; 18: 159–176.

[43] Akrich M . The description of technical objects. In: Bijker WE Law J (eds) Shaping technology/building society: Studies in sociotechnical change. London: MIT press, 1992, pp.205–224.

[44] Mol A Moser I Pols J . Care: putting practice into theory. In: Mol A Moser I Pols J (eds) Care in practice: on tinkering in clinics, homes and farms. Bielefeld: Transcript Verlag, 2010, pp.7–26.

[45] Latour B . We have never been modern. Harvard: Harvard University Press, 2012.

[46] de Boer B . Explaining multistability: Postphenomenology and affordances of technologies. AI Soc 2021; 3: 1–11.

[47] Husserl E . The essential Husserl: basic writings in transcendental phenomenology. Indiana: Indiana University Press, 1999.

[48] Orlikowski WJ Scott SV . 10 Sociomateriality: challenging the separation of technology, work and organization. Acad Manage Ann 2008; 2: 433–474.

[49] Barad K . Meeting the universe halfway: quantum physics and the entanglement of matter and meaning. Durham: Duke University Press, 2007.

[50] Ali M Zhou L Miller L , et al. User resistance in IT: a literature review. Int J Inf Manage 2016; 36: 35–43.

[51] Greenhalgh T Wherton J Sugarhood P , et al. What matters to older people with assisted living needs? A phenomenological analysis of the use and non-use of telehealth and telecare. Soc Sci Med 2013; 93: 86–94. 23906125 10.1016/j.socscimed.2013.05.036

[52] Ziebland S Hyde E Powell J . Power, paradox and pessimism: on the unintended consequences of digital health technologies in primary care. Soc Sci Med 2021; 289: 114419. 34619631 10.1016/j.socscimed.2021.114419

[53] Golay D . An invisible burden: an experience-based approach to nurses’ daily work life with healthcare information technology. Uppsala, Sweden: Uppsala University, 2019.

[54] Halcomb E Smyth E McInnes S . Job satisfaction and career intentions of registered nurses in primary health care: an integrative review. BMC Fam Pract 2018; 19: 1–14. 30086722 10.1186/s12875-018-0819-1 PMC6081816

[55] Bisht NS Trusson C Siwale J , et al. Enhanced job satisfaction under tighter technological control: the paradoxical outcomes of digitalisation. New Technol Work Employ 2021; 3. 10.1111/ntwe.12222.

[56] Jasanoff S Kim S-H . Dreamscapes of modernity. *Sociotechnical imaginaries and the fabrication of power Chicago* 2015.

[57] Villumsen S Faxvaag A Nøhr C . Development and progression in danish eHealth policies: towards evidence-based policy making. Studies in health technology and informatics 2019; 264: pp.1075–1079. 10.3233/SHTI190390. 31438090

[58] Wadhwa M. National eHealth Authority (NeHA). 2020.

[59] Warth LL Dyb K . Ehealth initiatives; the relationship between project work and institutional practice. BMC Health Serv Res 2019; 19: 1–12. 31340819 10.1186/s12913-019-4346-0 PMC6657135

[60] Andargoli AE . Ehealth in Australia: a synthesis of thirty years of eHealth initiatives. Telemat Inform 2021; 56: 101478.

[61] Socialdepartementet. Nationell IT-strategi för vård och omsorg [National IT strategy for health- and social care]. In: Socialdepartementet, (ed.). Stockholm 2006.

[62] Socialdepartementet. Nationell eHälsa – strategin för tillgänglig och säker information inom vård och omsorg. In: Socialdepartementet, (ed.). Västerås 2010.

[63] Socialdepartementet SKoL. Vision e-hälsa 2025 – gemensamma utgångspunkter för digitalisering i socialtjänst och hälso- och sjukvård. In: Socialdepartementet, (ed.). Stockholm 2016.

[64] Hellberg S Johansson P . eHealth strategies and platforms–The issue of health equity in Sweden. Health Policy Technol 2017; 6: 26–32.

[65] Longhurst R . Semi-structured interviews and focus groups. Key Methods in Geography 2003; 3: 143–156.

[66] Etikan I Musa SA Alkassim RS . Comparison of convenience sampling and purposive sampling. Am J Theor Appl Stat 2016; 5: 1–4.

[67] Goodman LA . Snowball sampling. Ann Math Stat 1961; 32: 148–170.

[68] Creswell JW Hanson WE Clark Plano VL , et al. Qualitative research designs: selection and implementation. Couns Psychol 2007; 35: 236–264.

[69] Kallio H Pietilä AM Johnson M , et al. Systematic methodological review: developing a framework for a qualitative semi–structured interview guide. J Adv Nurs 2016; 72: 2954–2965. 27221824 10.1111/jan.13031

[70] Elo S Kyngäs H . The qualitative content analysis process. J Adv Nurs 2008; 62: 107–115. 18352969 10.1111/j.1365-2648.2007.04569.x

[71] Walsham G . Doing interpretive research. Eur J Inf Syst 2006; 15: 320–330.

[72] Daymon C Holloway I . Qualitative research methods in public relations and marketing communications. London: Routledge, 2010.

[73] Braun V Clarke V . One size fits all? What counts as quality practice in (reflexive) thematic analysis? Qual Res Psychol 2020; 18(3): 1–25.

[74] Clarke V Braun V . Thematic analysis. In: Encyclopedia of critical psychology. Bristol: Springer, 2014, pp.1947–1952.

[75] Eisenhardt KM . Building theories from case study research. Acad Manage Rev 1989; 14: 532–550.

[76] Huberman AM Miles MB . Data management and analysis methods. 1994.

[77] Yin RK . The case study crisis: some answers. Adm Sci Q 1981; 26: 58–65.

[78] Leininger M . Evaluation criteria and critique. Critical Issues in Qualitative Research Methods 1994; 95: 351–367.

[79] Piras EM Miele F . On digital intimacy: redefining provider–patient relationships in remote monitoring. Sociol Health Illn 2019; 41: 116–131. 31599992 10.1111/1467-9566.12947

[80] Nimmon L Stenfors-Hayes T . The “handling” of power in the physician-patient encounter: perceptions from experienced physicians. BMC Med Educ 2016; 16: 1–9. 27091146 10.1186/s12909-016-0634-0 PMC4835893

[81] Winner L . Do artifacts have politics? Daedalus 1980; 109: 121–136.

[82] Fairman J D’Antonio P . Virtual power: gendering the nurse–technology relationship. Nurs Inq 1999; 6: 178–186. 10795271 10.1046/j.1440-1800.1999.00032.x

[83] Holgersson C Östlund B . Invisible workers: on digitalisation in home care work from a gender and technology perspective. In: Gendered norms at work. Stockholm, Sweden: Springer, 2021, pp.105–119.

[84] Jackson J Anderson JE Maben J . What is nursing work? A meta-narrative review and integrated framework. Int J Nurs Stud 2021; 122: 103944. 34325358 10.1016/j.ijnurstu.2021.103944

[85] Shaw SE Hughes G Hinder S , et al. Care organising technologies and the post-phenomenology of care: an ethnographic case study. Soc Sci Med 2020; 255: 112984. 32315872 10.1016/j.socscimed.2020.112984 PMC7262591

[86] Torenholt R Tjørnhøj-Thomsen T . ‘Is this something I should be worried about?’: a study of nurses’ recontextualisation work when making clinical decisions based on patient reported outcome data. Soc Sci Med 2022; 294: 114645. 35051741 10.1016/j.socscimed.2021.114645

[87] Dudhwala F Larsen LB . Recalibration in counting and accounting practices: dealing with algorithmic output in public and private. Big Data Soc 2019; 6: 2053951719858751.

[88] Schwennesen N . Algorithmic assemblages of care: imaginaries, epistemologies and repair work. Sociol Health Illn 2019; 41: 176–192. 31599986 10.1111/1467-9566.12900

[89] Jhala M Menon R . Examining the impact of an asynchronous communication platform versus existing communication methods: an observational study. BMJ Innov 2021; 7: 68–74. 10.1136/bmjinnov-2019-000409 PMC7808296 33479571

[90] Hoeyer K Wadmann S . ‘Meaningless work’: how the datafication of health reconfigures knowledge about work and erodes professional judgement. Econ Soc 2020; 49: 433–454.

[91] Latour B Porter C . Aramis, or, the love of technology. Cambridge, MA: Harvard: Harvard University Press, 1996.

[92] Callon M . Some elements of a sociology of translation: domestication of the scallops and the fishermen of St Brieuc Bay. Sociol Rev 1984; 32: 196–233

[93] Woolgar S Cooper G . Do artefacts have ambivalence: Moses’ bridges, winner's bridges and other urban legends in S&TS. Soc Stud Sci 1999; 29: 433–449.

